# Silencing of Taxol-Sensitizer Genes in Cancer Cells: Lack of Sensitization Effects

**DOI:** 10.3390/cancers7020824

**Published:** 2015-06-16

**Authors:** Shang-Lang Huang, Chuck C.-K. Chao

**Affiliations:** 1 Department of Biochemistry and Molecular Biology, College of Medicine, Chang Gung University, Taoyuan 333, Taiwan; E-Mail: shanglang@mail.cgu.edu.tw; 2 Graduate Institute of Biomedical Sciences, College of Medicine, Chang Gung University, Taoyuan 333, Taiwan; 3 Department of Medical Research and Development, Chang Gung Memorial Hospital, Taoyuan 333, Taiwan

**Keywords:** RNA silencing, synthetic lethality, taxol, taxol-sensitizer loci

## Abstract

A previous genome-wide screening analysis identified a panel of genes that sensitize the human non-small-cell lung carcinoma cell line NCI-H1155 to taxol. However, whether the identified genes sensitize other cancer cells to taxol has not been examined. Here, we silenced the taxol-sensitizer genes identified (acrbp, atp6v0d2, fgd4, hs6st2, psma6, and tubgcp2) in nine other cancer cell types (including lung, cervical, ovarian, and hepatocellular carcinoma cell lines) that showed reduced cell viability in the presence of a sub-lethal concentration of taxol. Surprisingly, none of the genes studied increased sensitivity to taxol in the tested panel of cell lines. As observed in H1155 cells, SKOV3 cells displayed induction of five of the six genes studied in response to a cell killing dose of taxol. The other cell types were much less responsive to taxol. Notably, four of the five inducible taxol-sensitizer genes tested (acrbp, atp6v0d2, psma6, and tubgcp2) were upregulated in a taxol-resistant ovarian cancer cell line. These results indicate that the previously identified taxol-sensitizer loci are not conserved genetic targets involved in inhibiting cell proliferation in response to taxol. Our findings also suggest that regulation of taxol-sensitizer genes by taxol may be critical for acquired cell resistance to the drug.

## 1. Introduction

Paclitaxel (taxol) and taxanes are microtubule-stabilizing agents that act primarily by interfering with spindle microtubules, causing cell cycle arrest and apoptosis. These drugs are used for the treatment of non-small-cell lung cancer (NSCLC) and ovarian cancer, among others. However, their therapeutic usefulness is limited by the phenomenon of acquired chemoresistance [1,2]. Drug resistance is associated with several membrane transporters of the ATP-binding cassette (ABC) and solute carrier (SLC) families. Glycoprotein P (P-gp, multidrug resistance protein 1, or MDR1), which is encoded by the abcb1 gene, is probably the most important ABC protein involved in this process [3,4,5]. P-gp functions as a drug efflux pump that actively removes around 20 different cytostatic drugs from cancer cells. In addition, the influx carriers SLC transporters [6] are often down-regulated in chemoresistant cells [7,8,9]. Taxol disrupts microtubules by binding to their interior surface [10]. Altered expression of microtubule-associated proteins (MAPs) (such as MAP4, stathmin, and tau) may be useful to identify cancer patients who may show recurrence and those most likely to benefit from taxol treatment [2]. Other biomarkers of taxol chemoresistance include specific checkpoint proteins including BRCA1 and the spindle assembly checkpoint proteins MAD2, BUBR1, synuclein-gamma, and aurora A.

Combining a high-throughput cell-based screening platform (one-well/one-gene) with a genome-wide library of chemically synthesized small interfering RNAs, Whitehurst and colleagues identified a panel of 87 genes that represent major focal points of the response of the human non-small-cell lung cancer (NSCLC) line NCI-H1155 to taxol [11]. Several of these gene products sensitize lung cancer cells to taxol at concentrations 1000 fold lower than otherwise required for a significant response. The effects of a panel of genes representing six functionally diverse groups from the “high-confidence” hit list have been verified [11]. This panel includes ACRBP (cancer/testis antigen or CT-antigen) [12], PSMA6 (an alpha type subunit of the 20S proteasome) [13], TUBGCP2 (microtubule-related c-TuRC protein) [14], HS6ST2 (a heparin sulphate transferase which is significantly overexpressed in ovarian and lung tumor tissues ([15]; White group, unpublished observations), ATP6V0D2 (channel protein and a vacuolar ATPase subunit expressed from a locus amplified in several lung cancer lines; [16]), and FGD4 (a CDC42 activator of the Ras family; [17]). These findings are attractive as they may lead to important improvements of existing cancer therapies. However, the effects of the taxol-sensitizer genes identified have not been investigated in other cancer types.

Considering that taxanes are important chemotherapeutic agents for the treatment of various cancers, we examined the role of representative taxol-sensitizer genes in ten types of cancer cell lines, including four non-small-cell lung carcinomas (NSCLCs): H1155 (neuroendocrine), H1299 (large cell), H520 (squamous cell), and H661 (large cell). Given the existence of several upregulated and downregulated pathways shared between cancer cells originating from different organs, we hypothesized that sensitizer genes identified in lung cancer cells may also produce sensitizing effects in other cancer cells. Our results indicate that the previously identified taxol-sensitizer genes are not conserved genetic targets that modulate cell proliferation in all taxol-treated cancer cells. We analyzed the regulation of these genes in various cell types and discuss possible reasons for the lack of sensitization effects observed in the cancer cell lines tested. Our results highlight possible pitfalls associated with the extrapolation of experimental data to other cell line models in chemoresistance studies.

## 2. Materials and Methods

### 2.1. Cell Lines and Reagents

NSCLC (H1155, H1299, H520, H661), hepatocellular carcinoma (Huh7, Hep3B, HepG2), cervical (HeLa), ovarian carcinoma (SKOV3; American Type Culture Collection, Rockville, MD, USA), and lymphoblastoma (HOB1) cell lines [18] were grown in a 1:1 mixture of DMEM/nutrient F-12 Ham (Life Technologies, Grand Island, NY, USA) supplemented with 1% (*w/v*) penicillin/streptomycin and 10% (*v*/*v*) fetal bovine serum (FBS) at 37 °C in a humidified atmosphere containing 5% CO_2_. The chemotherapeutic drugs used included taxol, vincristine, and cisplatin (Bristol-Myers Squibb, New York, NY, USA). Unless indicated otherwise, chemicals were purchased from Sigma-Aldrich (St. Louis, MO, USA). All reagents were used according to the instructions provided by the supplier.

### 2.2. Drug-Resistant Cell Lines

The SKOV3-derived taxol-resistant ovarian cancer (SKOV3/Tx600) [19], HeLa-derived cisplatin-resistant (HeLa/R3) [20], and HOB1-derived vincristine-resistant lymphoblastoma cell lines (HOB/VCR) [18] were prepared from the parental cell lines by administrating the drug in a dose-escalation manner. Drug-resistant cell lines were maintained in selective medium containing the drug concentration used for initial selection of resistance. The cells were cultured in drug-free medium for one week before the experiments. Periodic evaluation of half-maximal inhibitory concentrations (IC_50_) confirmed that the drug-resistance phenotype was stable for at least two months in a drug-free medium.

### 2.3. Cell Viability Assay

Cell viability was determined using the MTT [3-(4,5-dimethylthiazol-2-yl)-2,5-diphenyl-2H-tetrazolium bromide] colorimetric *in vitro* assay as previously described [19]. One hundred μL of cells was seeded at a density of 3 × 10^4^ cells/mL in 96-well microplates. Cells were exposed to taxol in culture medium at 37 °C for 72 h. Twenty μL of MTT solution (5 mg/mL in PBS) was added to each well, prior to incubation for 4 h. Optical density (OD) of the purple formazan product was measured at a wavelength of 540 nm using a spectrophotometer. The 50% inhibitory concentrations (IC_50_) of cell proliferation or cell viability were defined as the levels that respectively cause 50% reduction in cell viability *versus* the DMSO-treated control.

### 2.4. Quantitative PCR Analysis

Total RNA was extracted with the Trizol reagent (Life Technologies) as previously described [21]. RNA concentrations were assessed using a spectrophotometer, and only the samples with a A_260_/A_280_ ratio between 1.9 and 2.2 were used. Real-time quantitative PCR was performed on total RNA as before [22]. All unknown samples and controls were done in triplicate. Relative quantification was calculated using the ∆∆Ct method and normalized against GAPDH. Namely, the ∆Ct for each candidate was calculated as ∆Ct (candidate) = [Ct (candidate) − Ct (GAPDH)]. The relative abundance of the candidate gene X was shown as 2^∆Ct(X) − ∆Ct(GAPDH)^.

The primer pairs for PCR were as follows: acrbp (forward, CTGAAGTCTCACCCACCACGAT, reverse, TGGAAGGTCTGGCGTTCTG), atp6v0d2 (forward, GCCTGGTTCGAGGATGCA, reverse, TTCAGGTCTTCTAGGGTCTCACACT), fgd4 (forward, ACTTTGCAGCATCACATGCTAGA, reverse, GAGGCAATTTCCTTAGATAGTCCTTAAG), hs6st2 (forward, TGGGTCAGAAGAAATG CACTTG, reverse, CCAGCCCGTGGAGAACCT), psma6 (forward, GTTGTGTGATGACCGGAAT GAC, reverse, GTATTTCCAGTTAGCTGCCTCATAGC), and tubgcp2 (forward, CAGGAGGATTA CAACGACAAGTACTG, reverse, GCCATTTTCTGCAGGAAGGA).

### 2.5. Knockdown Assay

Knockdown of candidate genes was performed using commercially-available pLKO.1 plasmids expressing shRNA (National RNAi Core Facility, Academia Sinica, Taipei, Taiwan) as described before [9]. Luciferase shRNA (TRCN0000072244) was used as a negative control. Specific shRNA knockdown clones were selected for cell viability assay using puromycin. shRNA plasmids encoding genes highly overexpressed in taxol-resistant cells were selected and used in the present study. Both shRNA clone ID and target sequence were included: acrbp (TRCN0000115844, GTACCCAAACTACTGTTCCTT), atp6v0d2 (TRCN 0000043519, CCAGACTACTGATTATGGTAA), fgd4 (TRCN0000048233, CCATGAGATGAAGGAGACTAA), hs6st2 (TRCN0000036299, GCCTCTAGTGTAGAGATCAAT), psma6 (TRCN0000022369, GTAACAACAAACCAACATCAT), and tubgcp2 (TRCN0000139732, CCAGGAGGATTACAACGACAA). Knockdown efficiency was calculated by dividing the RNA level of cells expressing control luciferase shRNA by the RNA level of cells expressing target shRNA.

### 2.6. Statistical Analysis

Data were reported as mean values ± standard deviation (SD). Three independent experiments were performed unless indicated otherwise. Statistical significance (*p* value) was calculated using a two-tailed Student’s *t* test for single comparison.

## 3. Results

### 3.1. Sensitization of H1155 Cells to Taxol Following Silencing of Chemosensitizer Genes

**Figure 1:**
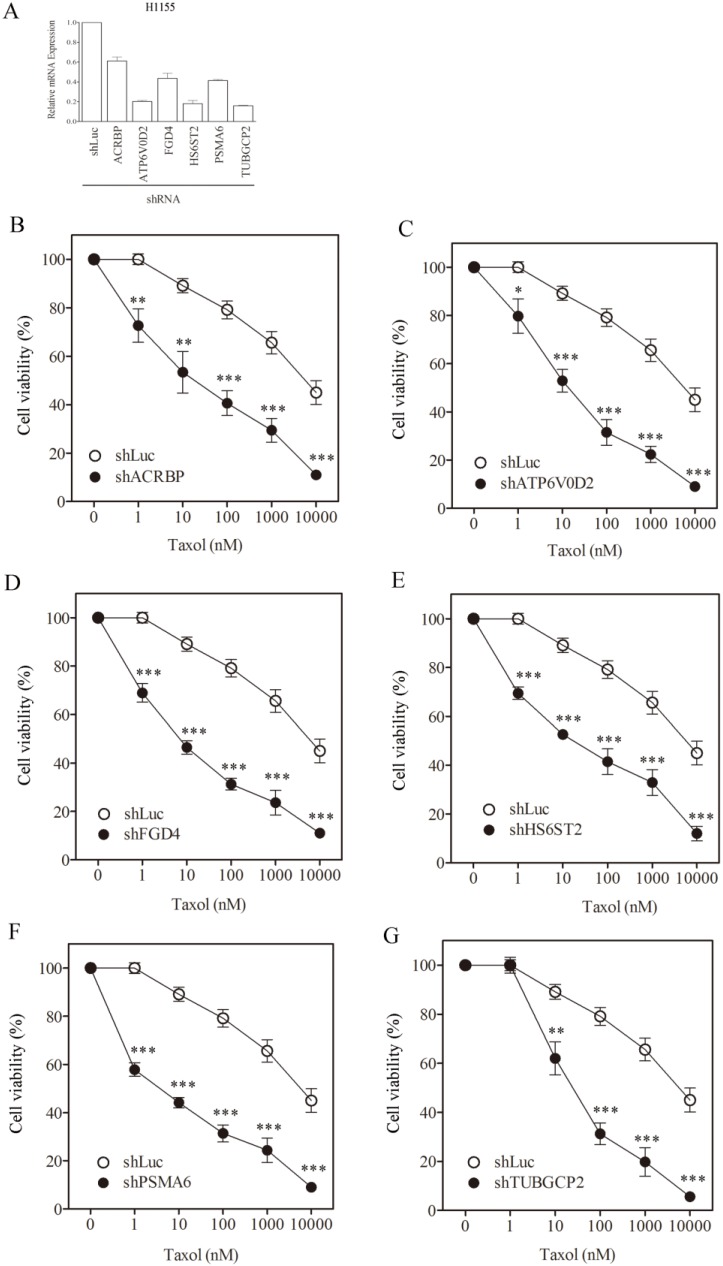
Sensitization of H1155 cells to taxol following silencing of chemosensitizer genes. (**A**) Silencing efficiency of representative taxol-sensitizer loci using shRNA in H1155 cells. Cell viability of H1155 cells against taxol treatment following silencing of acrbp (**B**); atp6v0d2 (**C**); fgd4 (**D**); hs6st2 (**E**); psma6 (**F**); and tubgcp2 (**G**). shLuc treated cells were used as control. All experiments reported in this study were performed in triplicate.

**Figure 2:**
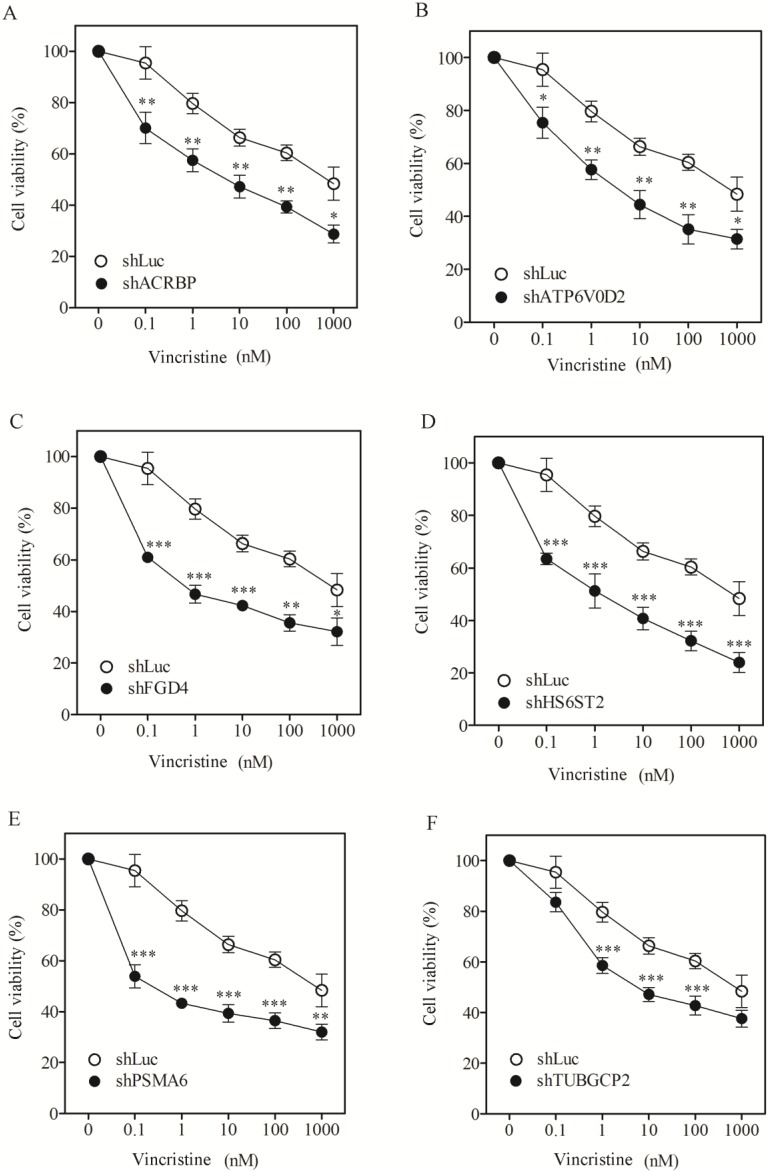
Sensitization of H1155 cells to vincristine after silencing of chemosensitizer genes. Cell viability of H1155 cells against vincristine following silencing of acrbp (**A**); atp6v0d2 (**B**); fgd4 (**C**); hs6st2 (**D**); psma6 (**E**); and tubgcp2 (**F**). shLuc treated cells were used as control. The experiments were performed in triplicate.

**Figure 3:**
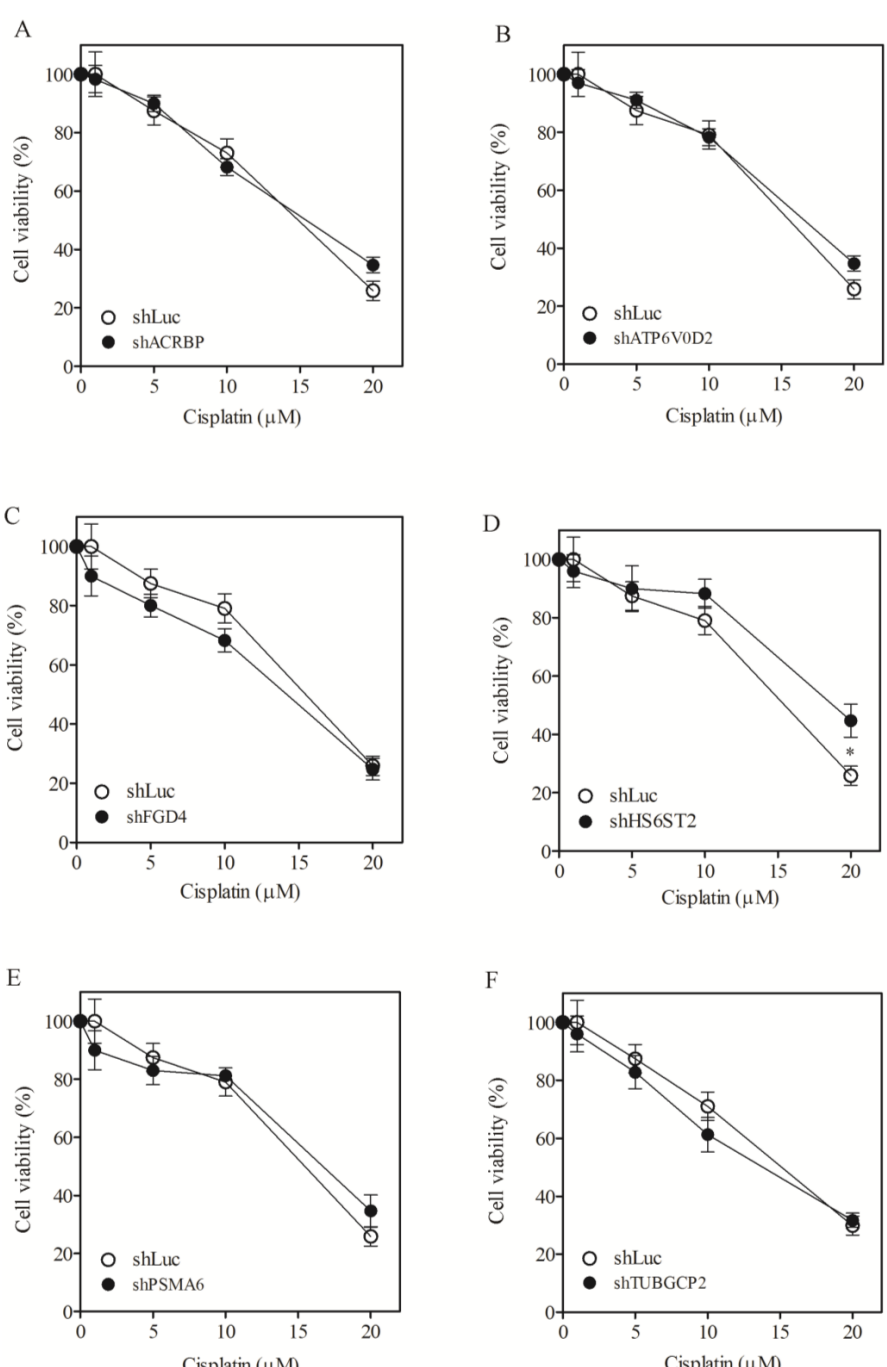
Lack of sensitization to cisplatin following silencing of chemosensitizer genes in H1155 cells. Cell viability of H1155 cells against cisplatin following silencing of acrbp (**A**); atp6v0d2 (**B**); fgd4 (**C**); hs6st2 (**D**); psma6 (**E**); and tubgcp2 (**F**). shLuc treated cells were used as control.

To assess the role of taxol-sensitizer genes, we silenced six of them using shRNA in the H1155 cell line (the cell line initially used to identify the taxol-sensitizer genes; [11]). The silencing efficacy of these genes ranged between 50% to 80%, except for ACRBP which showed 40% inhibition (Figure 1A). Under these silencing conditions, cell viability was determined following treatment with taxol at various concentrations. Silencing of the selected genes sensitized H1155 cells not only to taxol (Figure 1B–G) but also to vincristine (Figure 2A–F). However, none of the gene silencing performed sensitized H1155 cells to cisplatin (Figure 3A–F).

We determined the IC_50_ of the cells following silencing treatment (Table 1). In order to quantify the level of sensitization, we calculated the ratio of IC_50_ of shLuc control cells divided by the IC_50_ of cells in which a gene had been silenced (Table 1, numbers in parentheses). Silencing FGD4 and PSMA6 produced a high level of sensitization to taxol, reaching 911- and 1273-fold, respectively. Silencing FGD4 and PSMA6 also produced high sensitization to vincristine (1105- and 2051-fold, respectively). Silencing of the other taxol-sensitizer genes also sensitized cells to taxol and vincristine by more than 100-fold. Nevertheless, none of the gene silencing trials could affect cell sensitivity to ciplatin. The extent of sensitization observed following silencing of these genes in our system was comparable to that reported previously [11]. These results support the notion that the genes under study are responsive specifically to microtubule-damaging drugs such as taxol [11].

**Table 1:** Drug concentrations that kill H1155 cells (IC50) and sensitization by silencing taxol-sensitizer loci.

	H1155, IC_50_ (SF)		
	Taxol (nM)	Vincristine (nM)	Cisplatin (µM)
shLuc	7817.1 ± 601	876.3 ± 61.4	14.9 ± 2.1
shACRBP	34.0 ± 1.6 (230.1)	7.5 ± 0.8 (116.1)	15.4 ± 1.1 (ND)
shATP6V0D2	22.1 ± 1.1 (354.1)	6.2 ± 0.4 (141.3)	16.5 ± 2.3 (ND)
shFGD4	8.6 ± 1.1 (911.4)	0.8 ± 0.0 (1105.2)	14.2 ± 1.0 (ND)
shHS6ST2	31.2 ± 2.9 (250.7)	2.1 ± 0.4 (419.0)	18.8 ± 3.4 (ND)
shPSMA6	6.1 ± 0.4 (1272.5)	0.4 ± 0.0 (2050.9)	16.7 ± 2.2 (ND)
shTUBGCP2	45.1 ± 5.6 (173.3)	7.8 ± 0.9 (112.9)	13.8 ± 0.9 (ND)

Numbers in parentheses represent SF calculated as the ratio of IC_50_ between control shLuc and shRNA treatment. ND: No difference in IC_50_ between control and specific gene knockdown.

### 3.2. Lack of Sensitization of Non-H1155 Cells to Taxol Following Silencing of Chemosensitizer Genes

**Figure 4:**
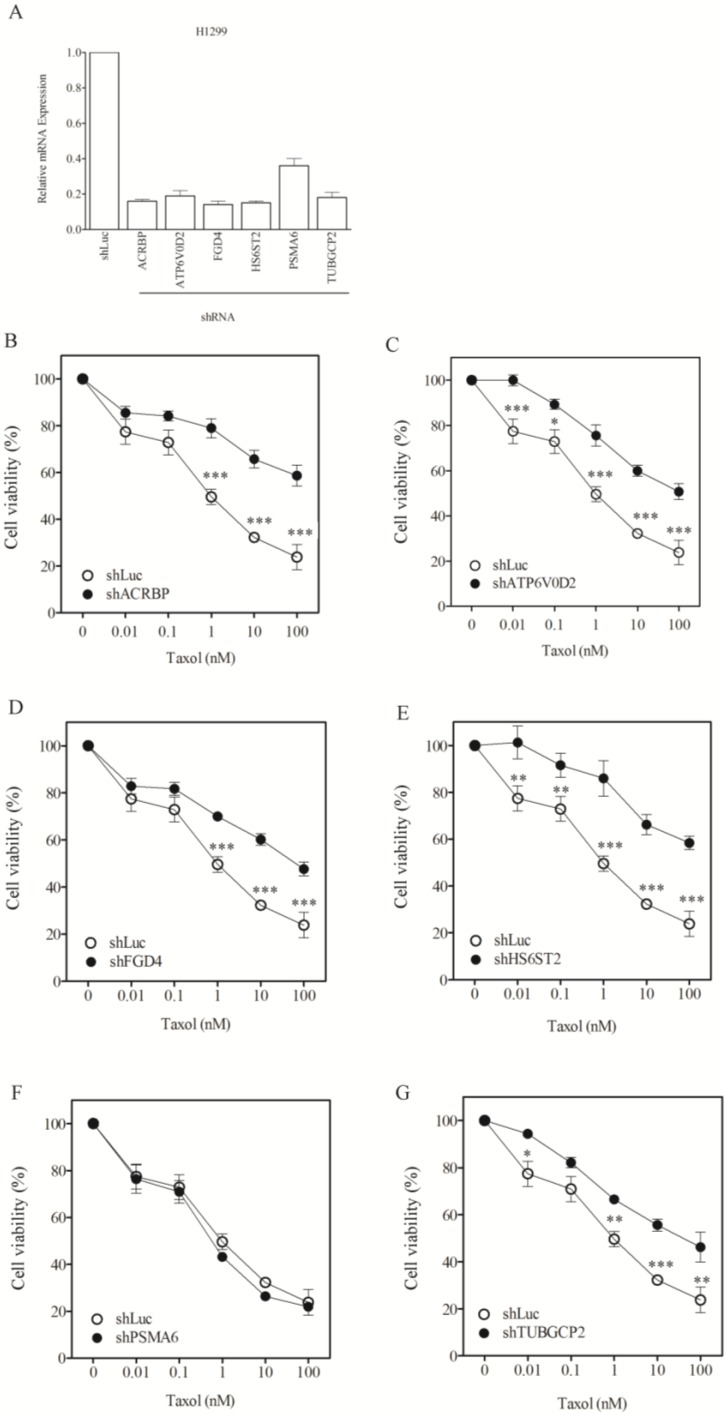
Lack of sensitization of H1299 cells to taxol by chemosensitizer loci silencing. (**A**) Silencing efficiency of representative taxol-sensitizer loci using shRNA in H12995 cells; Cell viability of H1299 cells against taxol following silencing of acrbp (**B**); atp6v0d2 (**C**); fgd4 (**D**); hs6st2 (**E**); psma6 (**F**); and tubgcp2 (**G**). shLuc treated cells were used as control.

**Figure 5:**
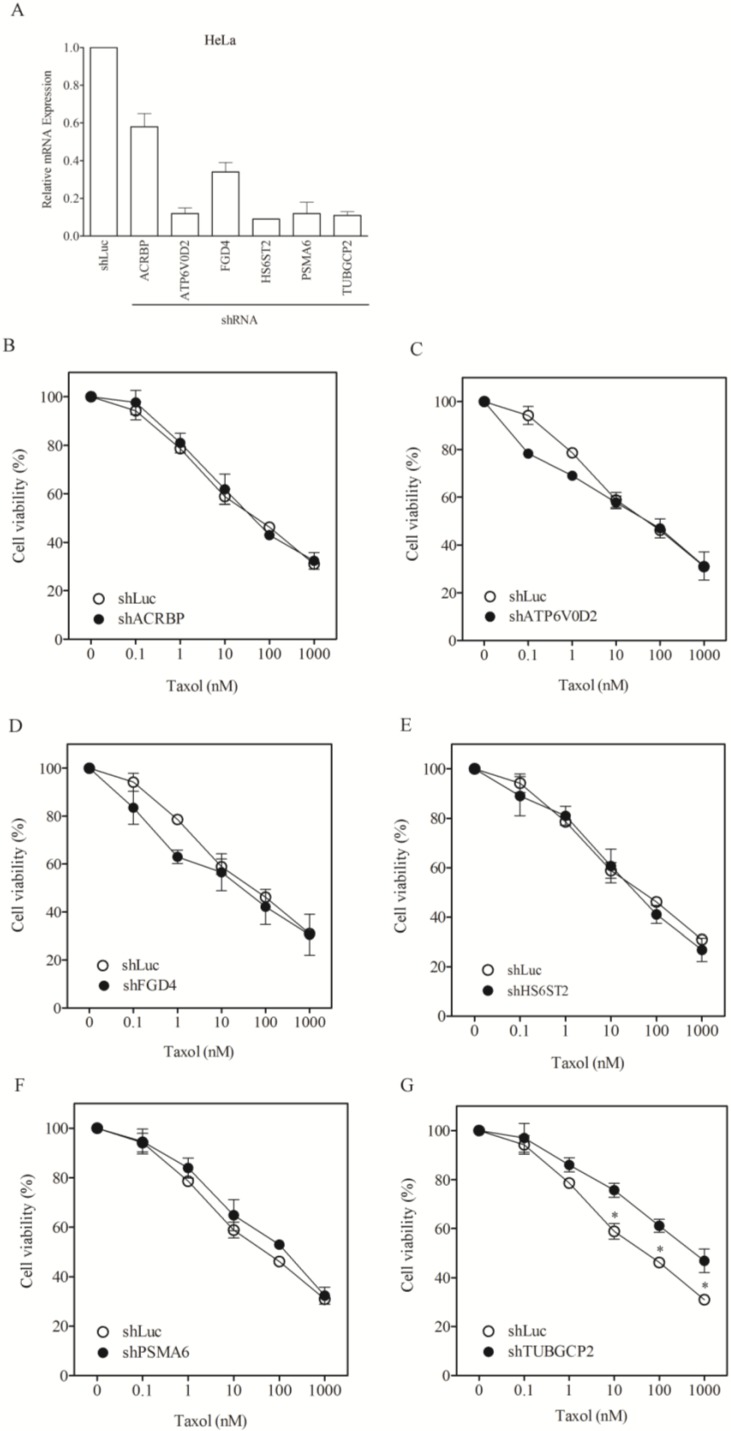
Lack of sensitization of HeLa cells to taxol by chemosensitizer loci silencing. (**A**) Silencing efficiency of representative taxol-sensitizer loci using shRNA in HeLa cells; Cell viability of HeLa cells against taxol following silencing of acrbp (**B**); atp6v0d2 (**C**); fgd4 (**D**); hs6st2 (**E**); psma6 (**F**); and tubgcp2 (**G**). shLuc treated cells were used as control.

**Figure 6:**
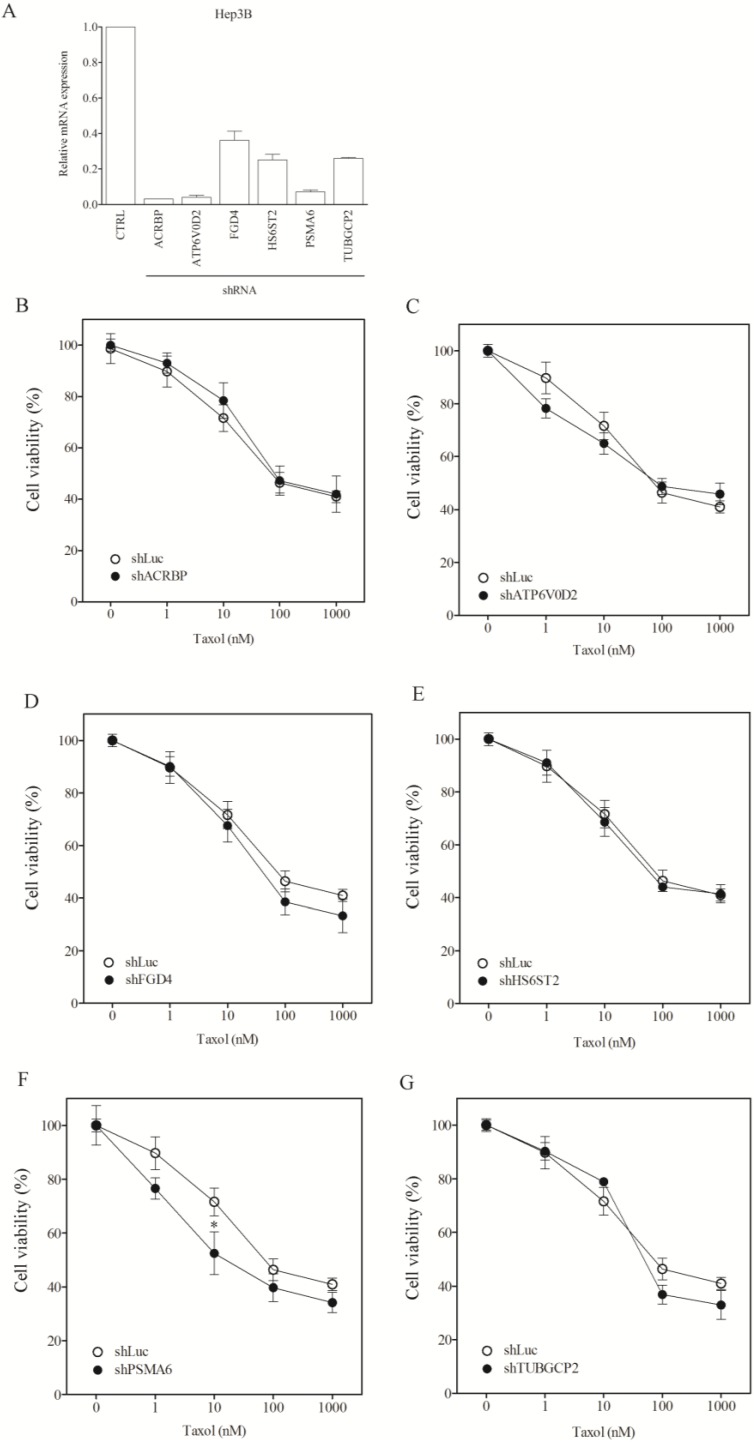
Lack of sensitization of Hep3B cells to taxol following silencing of chemosensitizer loci. (**A**) Silencing efficiency of representative taxol-sensitizer loci using shRNA in Hep3B cells; Cell viability of Hep3B cells against taxol following silencing of acrbp (**B**); atp6v0d2 (**C**); fgd4 (**D**); hs6st2 (**E**); psma6 (**F**); and tubgcp2 (**G**). shLuc treated cells were used as control.

To assess whether the modulatory role of the taxol-sensitizer genes in other cancer cells, we examined the response of nine other types of cancer cell lines, including lung (H1299, H520, H661), liver (Huh7, Hep3B, HepG2), cervical (HeLa), neuroblastoma (HOB1) and ovarian cancer cells (SKOV3). Silencing of the taxol-sensitizer genes produced no sensitization to taxol in these cell lines. In fact, silencing 5 of the 6 genes induced taxol resistance in H1299 cells (Figure 4B–G). These results were not due to poor gene silencing efficacy since inhibition over 60%–80% was observed (Figure 4A), compared to 40%–80% in H1155 cells (Figure 1A). Negative results were obtained for HeLa and Hep3B cells (Figure 5 and Figure 6, respectively). The level of sensitization to taxol in these three cancer cell types was calculated using the sensitization factor (SF) (Table 2), which indicated a lack of sensitization. Only a minimal sensitization effect (SF = 3.2) was detected for PSMA6 in Hep3B cells following silencing of this gene to a level over 90%. This modification appeared negligible by comparison with the high SF (1273) obtained following silencing of this gene at 60% in H1155 cells (Figure 1A and Table 1). These results indicate that the genes studied produce no sensitization effects in the cancer cells tested.

**Table 2:** Taxol concentrations that kill non-H1155 (H1299, HeLa and Hep3B) cells (IC_50_) and sensitization by silencing taxol-sensitizer loci.

	Taxol nM, IC_50_ (SF)		
	H1299	HeLa	Hep3B
shLuc	1.0 ± 0.1	72.7 ± 3.4	87.0 ± 8.1
shACRBP	656.4 ± 51.3 (0.00) *	66.4 ± 3.9 (1.10)	92.1 ± 5.6 (0.94) *
shATP6V0D2	150.3 ± 21.6 (0.01) *	75.0 ± 6.1.0 (0.96) *	92.7 ± 11.0 (0.94) *
shFGD4	83.0 ± 9.6 (0.01) *	50.8 ± 4.4 (1.43)	76.1 ± 6.1(1.14)
shHS6ST2	637.8 ± 71.2 (0.00) *	67.3 ± 9.1 (1.08)	78.1 ± 9.0 (1.11)
shPSMA6	0.8 ± 0.06 (1.25)	228.5 ± 13.9 (0.32) *	27.4 ± 1.2 (3.18)
shTUBGCP2	63.7 ± 8.1 (0.02) *	798.1 ± 64.4 (0.09) *	71.7 ± 4.3 (1.21)

Numbers in parentheses represent SF calculated as the ratio of IC_50_ between control shLuc and shRNA treatment. *: Higher IC_50_ than control shLuc.

### 3.3. Sensitive Response of Chemosensitizer Genes to Taxol in H1155 Cells

**Figure 7:**
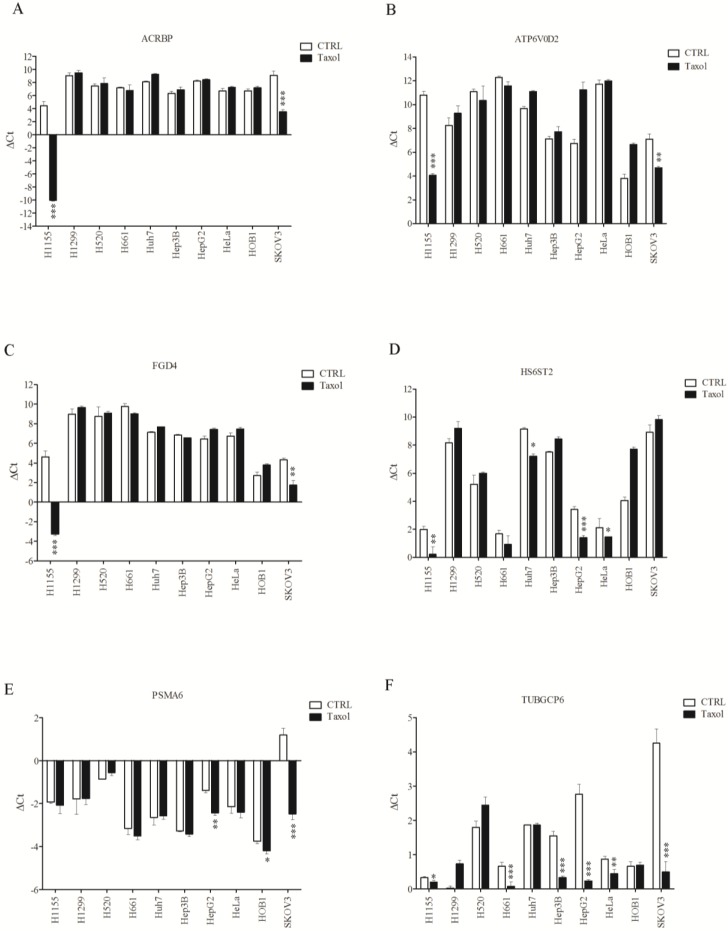
Sensitive response of chemosensitizer loci to taxol in H1155 and SKOV3 cells. The differential mRNA level of representative chemosensitizer genes, including acrbp (**A**); atp6v0d2 (**B**); fgd4 (**C**); hs6st2 (**D**); psma6 (**E**); and tubgcp2 (**F**) induced by taxol (1 nM) was quantified (see Materials and Methods for detail) in ten cancer cell lines. shLuc treated cells were used as control. (* *p* < 0.05, ** *p* < 0.01, *** *p* < 0.005).

The reason why the sensitization effects were only found in H1155 cells remains unclear. Based on our observations, the genes that are overexpressed in taxol-resistant cells were shown to be inducible by a low cytotoxic dose of the drug. We attempted to correlate the inducibility of the six taxol senitizer genes following taxol treatment with the overexpression of these genes in taxol-resistant cells. The RNA level of taxol-sensitizer genes in the ten cell lines was measured using qPCR following treatment with 1 nM of taxol (Figure 7). High induction of acrbp, atp6v0d2, and fgd4 was observed only in H1155 cells, and a moderate induction was also detected in SKOV3 cells (Figure 7A–C). No induction of these genes could be detected in the other cell lines tested. Induction of hs6st2 following taxol treatment was moderate in H1155 and HepG2 cells, while minor induction was observed in Huh7 and HeLa cells (Figure 7D). While psma6 was highly induced by taxol in SKOV3 cells and was moderately induced in HepG2 cells, this gene was not induced in H1155 cells (Figure 7E). Furthermore, tubgcp2 was slightly induced in H1155 cells, whereas it was highly induced in five of the other cell lines tested (H661, Hep3B, HepG2, HeLa, and SKOV3; Figure 7F). Notably, the tubgcp2 gene was highly responsive to taxol in the other five cell lines tested. It appears that the level of taxol-induced regulation of these genes (5 of 6) may be most significant in H1155 and SKOV3 cells.

### 3.4. Overexpression of Chemosensitizer Genes in Drug-Resistant Cells

**Figure 8:**
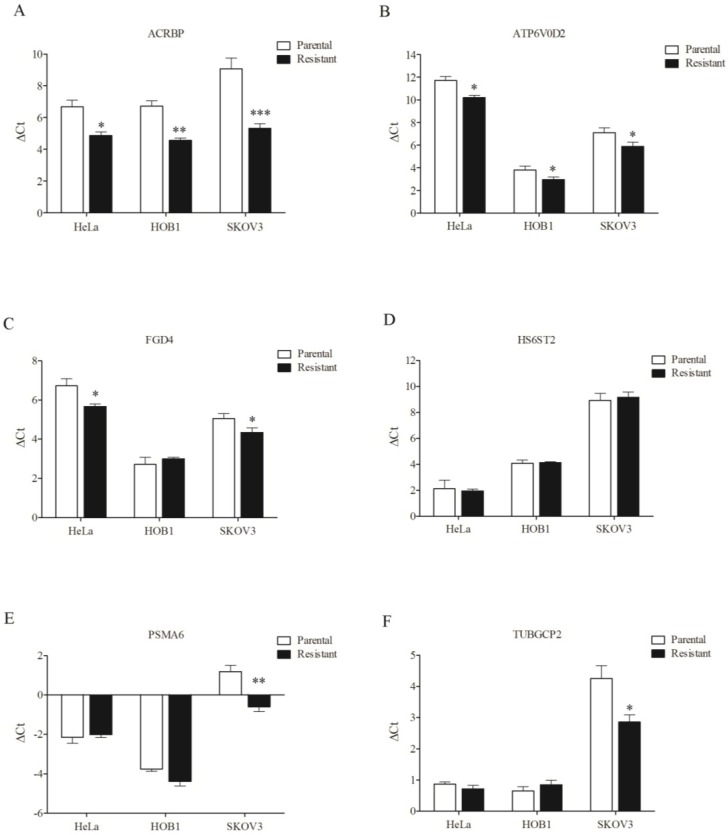
Overexpression of chemosensitizer genes in drug-resistant cells. mRNA level of representative chemosensitizer genes, including acrbp (**A**); atp6v0d2 (**B**); fgd4 (**C**); hs6st2 (**D**); psma6 (**E**); and tubgcp2 (**F**) was compared in paired isogenic cancer cell lines. The method for quantification of differential mRNA level is same as.

To assess whether the chemosensitizer genes are important for taxol resistance, we monitored their expression levels in drug-resistant cells. The level of acrbp and atp6v0d2 expression was upregulated in cisplatin-resistant HeLa, vincristine-resistant HOB1, and taxol-resistant SKOV3 cells (Figure 8A,B). fgd4 was upregulated in resistant HeLa and SKOV3 cells (Figure 8C). While hs6st2, psma6, and tubgcp2 were not upregulated in resistant HeLa and HOB1 cells, psma6 and tubgcp2 were upregulated in taxol-resistant SKOV3 cells (Figure 8D–F). These results indicate that five of six taxol-sensitizer genes are upregulated in taxol-resistant SKOV3 cells. We also noted that hs6st2 was the only gene among the six studied that was not downregulated in SKOV3 cells (Figure 7).

## 4. Discussion

In this study, we assessed the effects of silencing of taxol-sensitizer genes on the response of cancer cell lines to taxol. These genes were described earlier as being able to sensitize the H1155 cancer cell line to taxol, with sensitization levels at times reaching over 1000 fold [11]. Using shRNA designed to silence a panel of taxol-sensitizer genes in H1155 cells, we also observed high sensitization levels reaching 1000-fold or more for fgd4 and psma6. Although sensitization was less effective for the other genes, we still obtained sensitization levels over 100-fold in these cases. For unclear reasons, using antisense RNA to silence taxol-sensitizer genes in the cell lines tested produced extremely poor silencing efficiency in our hands. The differences in the levels of sensitization observed here and in the previous study that used antisense RNA [11] may be partly explained by different silencing efficacy. While cells were collected two days following oligonucleotide transfection in the previous study [11], the cells that we used were established by puromycin selection and they expressed shRNA for gene silencing. Still, the possibility that the different results obtained in these two studies may be due at least in part to differences in cell growth cannot be excluded.

**Figure A1:**
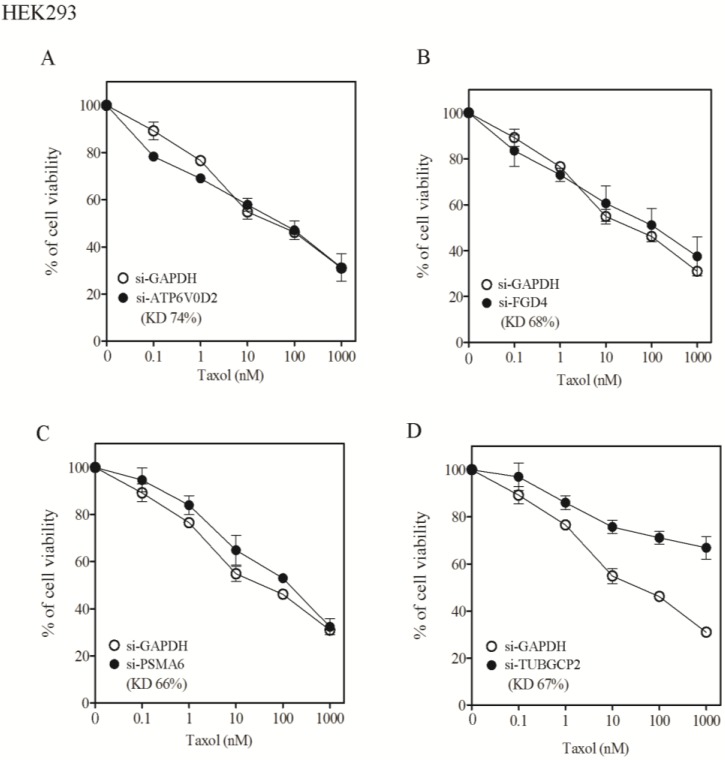
Lack of sensitization of HEK293 cells to taxol by chemosensitizer loci silencing. Cell viability of HEK293 cells against taxol following SiRNA silencing of *ATP6V0D2* (**A**); *FGD4* (**B**); *PSMA6* (**C**); and *TUBGCP2* (**D**). shGAPDH treated cells were used as control.

Silencing of these genes also sensitized cancer cells to vincristine in our experiments. On the other hand, the genes did not modify cell response to cisplatin, supporting the notion that the taxol-sensitizer genes are specific for cell response to microtubule-damaging agents [11]. However, powerful sensitization effects were noted only in H1155 cells, while no effect was observed in three other NSCLC cell lines. This observation is unlikely to be due to the silencing methods used since silencing of these genes in HEK293 cells using antisense RNA also failed to produce any sensitization effects (Appendix Figure A1). After performing a search of taxol-based treatments in The Cancer Genome Atlas (cBioportal.org), we found no correlation between the mRNA levels of “sensitizer” genes and complete or incomplete response to taxol treatment for advanced breast cancers (GSE22513). Similarly, no correlation was found for cancer-free and recurrent ovarian carcinoma patients treated with taxol. On the other hand, we observed that the “sensitizer” genes psma6 and tubgcp2 were overexpressed in hepatocellular carcinoma compared to normal cells (*P* = 0.02 for both genes). Based on this information, it appears that no solid clinical data has been obtained to support the function of these genes in taxol chemoresistance. Based on these results, we conclude that the effects of the taxol-sensitizer loci are not conserved throughout cancer cells.

Among the six taxol-sensitizer genes tested, four of them (acrbp, atp6v0d2, fgd4, and tubgcp6) were upregulated in response to taxol in both H1155 and SKOV3 cells. Except for tubgcp6, these genes were not upregulated in the other cancer cells tested. The tubgcp6 gene could be induced in six of ten cell lines, including five cell lines which were not sensitized to taxol following silencing of the gene. These results suggest that specific gene response to taxol may be required but not sufficient to produce a cell response to the drug. Previous studies by others have indicated that silencing of ACRBP (testis cancer antigen) or TUBGCP2 (microtubule-associated protein) did not affect cell viability but did enhance taxol-induced mitotic arrest in H1299 and H2126 cells (NSCLC, adenocarcinomas) [11]. The lack of change in viability in these NSCLC cells other than H1155 may reflect differences in the coupling between spindle assembly checkpoint machinery and apoptosis [23]. In addition, silencing of ACRBP or TUBGCP2 in NSCLC cell lines that lack a robust spindle assembly checkpoint [e.g., HCC366 (adenosquamous), HCC15 (squamous cell) or HCC4017 (adenocarcinoma)] was sufficient to induce accumulation of non-proliferating micro-nucleated cells, which are characteristic of taxol treatment [11]. These observations suggest that aberrant gene-expression programs may support fundamental biological mechanisms required for cancer cell proliferation. These observations may also explain, at least in part, the lack of sensitization of non-H1155 cells to taxol following silencing of taxol-sensitizer genes. It is possible that several genes may need to be silenced concomitantly in order to observe sensitizing effects, rather than single genes being silenced separately as reported here. In addition, some of these genes may not be involved in the response to taxanes in the cell lines studied here, especially for the genes involved in G2M checkpoint modulation and mitosis.

Expression of certain genes such as the taxol-sensitizers may be critical for the cells to overcome mitotic arrest following treatment with mitotoxins such as taxol. Differential response in the expression of these genes following treatment with taxol may contribute to differential mitotic progression by enhancing the robustness of the mitotic spindle apparatus. Prolonged activation of mitotic checkpoint is important for taxol-based therapy in various cancer cells. Lack of response of taxol-sensitizer genes to taxol in the non-H1155 cells tested is likely to be due to aberrant mitotic arrest and/or inefficient reduction of checkpoint and apoptotic activation. Silencing of the genes may sensitize cancer cells like H1155 to the drug only when the mitotic spindle apparatus is appropriately regulated and reduction of the apoptotic apparatus is successful. Whether these pathways are defective in non-H1155 cells is worthy of further investigation.

Notably, we found enhanced expression of five of six taxol-sensitizer genes in taxol-resistant ovarian cancer cells (SKOV3/Tx600). The only gene that was not upregulated in the resistant cells, hs6st2, was the same that was not inducible in the parental, sensitive SKOV3 cells. Two of the six genes studied (acrbp and atp6v0d2) were also upregulated in vincristine-resistant HOB1 cells. However, except for abcb1, the genes that were upregulated in vincristine-selected SKOV3 cells [24] were different from that found to be upregulated in taxol-selected SKOV3 [19], suggesting that the genes may play different roles in the development of resistance to the two drugs. This observation may partly explain the finding that more taxol-sensitizer genes are upregulated in taxol-resistance cells, while less are upregulated in vincristine-resistance cells. Unexpectedly, three of six genes (acrbp, atp6v0d2, and fgd4) that are taxol-associated were also minimally upregulated in cisplatin-resistant HeLa cells (Figure 8), suggesting that taxol-sensitizer genes may regulate cell response to genotoxins. A simple possible explanation is that cisplatin also induces mitotic arrest through DNA damage in HeLa cells, as reduced M-arrest was found in resistant cells [25]. These results suggest that upregulation of taxol-sensitizer loci may be important for the cells to acquire resistance to mitotoxins, specifically to taxol. In this context, most taxol-sensitizer genes are not upregulated during the development of taxol resistance. Although our findings of taxol-sensitizer genes could not be replicated in other cancer cells, the identification of these genes in H1155 cells is important to understand the mechanisms underlying cancer cell response to taxol, and it may provide a strategy to design synthetic therapy to overcome acquired taxol resistance.

## 5. Conclusions

Rational approaches to target genes that modulate drug response are now being considered in the pipeline of drug development for cancer therapy. Accumulating evidence suggests that many of these genes reflect states of dependency that are unique to cancer cells. Such dependency states can arise due to a strict dependency on a single oncogene in specific cancer cell line, or even to a non-oncogene. Identification of these “Achilles’ heels” within individual tumors remains an important advancement for the development of targeted therapies. High-throughput genome-wide RNA interference screening of cancer cell lines has started to reveal novel candidates for context-dependent therapeutic targets. Here, we examined the role of taxol-sensitizer genes identified previously using high-throughput screening and genome-wide RNA interference. Our results suggest that these genes are not consensus targets involved in chemoresistance in various cancer cells and do not function in modulating cancer cell proliferation in general. The reason for this apparent contradiction could be due to different cellular responses to the drug in the cell lines tested. Nevertheless, some taxol-sensitizer genes are associated with a sensitive response to the drug in an ovarian cancer cell line and are up-regulated in taxol-resistant variants. Findings concerning the various forms of cancer cell dependency to specific genes and the relevance of these processes in resistance to chemotherapeutic drugs may still lead to the development of more effective cancer therapies in the near future.

## Abbreviations

DMSO: dimethyl sulfoxide
FBS: fetal bovine serum
GAPDH: glyceraldehyde-3-phosphate dehydrogenase
MDR: multiple drug resistance
MTT: 3-(4,5-dimethylthiazol-2-yl)-2,5-diphenyltetrazolium bromide
PBS: phosphate-buffered saline
PCR: polymerase chain reaction
q-PCR: quantitative real-time polymerase chain reaction
shRNA: short-hairpin RNA
